# Alanine mutation of the catalytic sites of Pantothenate Synthetase causes distinct conformational changes in the ATP binding region

**DOI:** 10.1038/s41598-017-19075-2

**Published:** 2018-01-17

**Authors:** Bharati Pandey, Sonam Grover, Sukriti Goyal, Anchala Kumari, Aditi Singh, Salma Jamal, Jagdeep Kaur, Abhinav Grover

**Affiliations:** 1 Department of Biotechnology, Panjab University, Chandigarh, 160014 India; 2 Kusuma School of Biological Sciences, Indian Institute of Technology Delhi, New Delhi, 110016 India; 3 School of Biotechnology, Jawaharlal Nehru University, New Delhi, 110067 India; 4 Department of Bioscience and Biotechnology, Banasthali University, Tonk, Rajasthan 304022 India; 5 Department of Biotechnology, TERI University, VasantKunj, New Delhi, 110070 India

## Abstract

The enzyme Pantothenate synthetase (PS) represents a potential drug target in *Mycobacterium tuberculosis*. Its X-ray crystallographic structure has demonstrated the significance and importance of conserved active site residues including His44, His47, Asn69, Gln72, Lys160 and Gln164 in substrate binding and formation of pantoyl adenylate intermediate. In the current study, molecular mechanism of decreased affinity of the enzyme for ATP caused by alanine mutations was investigated using molecular dynamics (MD) simulations and free energy calculations. A total of seven systems including wild-type + ATP, H44A + ATP, H47A + ATP, N69A + ATP, Q72A + ATP, K160A + ATP and Q164A + ATP were subjected to 50 ns MD simulations. Docking score, MM-GBSA and interaction profile analysis showed weak interactions between ATP (substrate) and PS (enzyme) in H47A and H160A mutants as compared to wild-type, leading to reduced protein catalytic activity. However, principal component analysis (PCA) and free energy landscape (FEL) analysis revealed that ATP was strongly bound to the catalytic core of the wild-type, limiting its movement to form a stable complex as compared to mutants. The study will give insight about ATP binding to the PS at the atomic level and will facilitate in designing of non-reactive analogue of pantoyl adenylate which will act as a specific inhibitor for PS.

## Introduction

The causative agent of tuberculosis (TB) is *Mycobacterium tuberculosis* (Mtb), a major infectious bacterium which spreads through droplets in the air. TB is second only to HIV/AIDS as the major killer across the globe^1^. Multi-drug resistant *Mtb* is becoming a regular health problem especially in immuno-compromised individuals with HIV^2^. This form of TB is more difficult to treat and as a result has higher mortality rate. Because of this, the discovery of drugs targeting novel pathways such as the synthesis of pantothenate has become increasingly important. The World Health statistics report in 2014 stated that 9.6 million people were diagnosed with TB of which 1.5 million people died^3^.

*PanC* gene encodes Pantothenate Synthetase (PS; EC 6.3.2.1) responsible for producing pantothenate (vitamin B5), is a promising drug target owing to a few important reasons^4^. Firstly, it is present in all bacteria but absent in mammals, a key factor for the selective activity of drug molecule^5^. Secondly, Pantothenate is notable for its role in the synthesis of coenzyme A (CoA) and acyl carrier protein (ACP), essential components of fatty acid synthesis which maintain persistent growth and pathogenicity of the *M. tuberculosis*^6^. Lastly, Jacob *et al*., conducted research on a TB vaccine which compromised panC auxotrophs’ growth and virulence rigorously supporting the theory of functional necessity of Pantothenate Synthetase pathway and enhancing its attractiveness as a potential antimicrobial target^7^. PS proceeds by Bi Uni Uni Bi Ping Pong kinetic reactions; it catalyzes the ATP dependent condensation of pantoate with beta-alanine via a pantoyl adenylate intermediate as follows:

1 $${\rm{ATP}}+({\rm{R}}) \mbox{-} {\rm{pantoate}}+\mathrm{beta}{\rm{ \mbox{-} }}\mathrm{alanine}={\rm{AMP}}+{\rm{diphosphate}}+({\rm{R}}) \mbox{-} {\rm{pantothenate}}({\rm{vitamin}}\,{\rm{B}}5)$$

The Mg^2+^ dependent reaction involves two sequential steps; initially binding of ATP with pantoate, leads to formation of pantoyl adenylate and release of pyrophosphate. Second step involves nucleophilic attack by β-alanine on the active carbonyl group of pantoyl adenylate and subsequent release of AMP and pantothenate^8^.

PS has been cloned, expressed, purified and well-characterized from *M. tuberculosis* and *Escherichia coli* and their X-ray crystallized three dimensional structure are available in PDB (PDB ID: 1MOP and 1IHO)^9,10^. In addition, PS from *M. tuberculosis* (MtPS) bound with various substrate such as ATP, AMPCPP, pantoate, β-alanine and pantoyl adenylate have been resolved and submitted to PDB (PDB ID: 2A84, 2A7X, 2A86, 2A88)^4^. It is member of cytidylyl transferase family of enzyme and a homodimer with a subunit molecular mass of 33 kDa and 290 amino acid residue^6^. The structure composed of two domains with 3 sheets, 10 strands, and 16 helices and linked by numerous loops and turns.

Despite the availability of structural information and pathway for PS, until now no FDA approve drug against MtbPS have been reported. Therefore, several experiments have been conducted in the past in order to identify potential inhibitors against MtbPS, using high-throughput screening (HTS) against ZINC database and commercial library, fragment-based approach, and pharmacophore based screening^11–13^. It was found that PS is conserved across bacterial and eukaryotic species and also showed high degree of similarity of active site between PS of *Escherichia.coli* and Mtb^8^. Zheng *et al*.^6^, evaluated the functional roles and catalytic properties of the strictly conserved residues of the *M. tuberculosis* Pantothenate Synthetase (MtbPS) by constructing six alanine mutants. Alanine substitution at H44, H47, N69, Q72, and K160 residues in MtbPS led to inhibition of enzyme activity by 1000-fold, signifying the importance of these residues in the formation of the pantoyl adenylate intermediate.Furthermore, Isothermal titration microcalorimetry analysis confirmed that substitution at H47 and K160 for alanine leads to decrease affinity of the PS for ATP. The dynamics behavior and structural mechanism for differences in the MtbPS enzyme activity is still unveiled.

The estimation of structural changes due to point mutation will provide comprehensive account of the enzyme activity. In this study, we systematically investigated the conformational modification due to alanine substituted conserved active site residues using long term molecular dynamics (MD) simulations. Subsequently, residue network analysis, binding energy estimation (MM-GBSA), principle component analysis (PCA), and free energy landscape (FEL) analysis were also carried out to describe the dynamics behavior of alanine mutants.

## Results

The present study was intended to explore, how substitution in the highly conserved active site residues (H47, H44, N69, Q72, K160 and Q164) in PS protein from *M. tuberculosis*, leads to remarkable reduction in the catalytic activity of PS. The extensive computational approach was used to gain insight into the effect of alanine substitution on the binding of ATP with PS.

### Molecular dynamics simulation of the wild-type and alanine mutants in unbound form

The availability of the three dimensional structure of MtbPS enabled us to investigate dynamics of enzyme and its affinity for substrate in detail. The conserved active site residue H44, H47, N69, Q72, K160, and Q164 mainly belongs to the helix and loop region (Figure S1(A)). All polar charged (H44, H47 and K160) and polar uncharged (N69, Q72 and Q164) amino acid were replaced by non-polar, aliphatic (alanine) residue respectively. The crystal structure of MtbPS (PDB ID: 1MOP) was used to create alanine mutants (H44A, H47A, N69A, Q72A, K160A and Q164A) and subjected to 50 ns MD simulations to study comparative conformation dynamics of the wild-type and mutants in unbound form.

**Figure 1:**
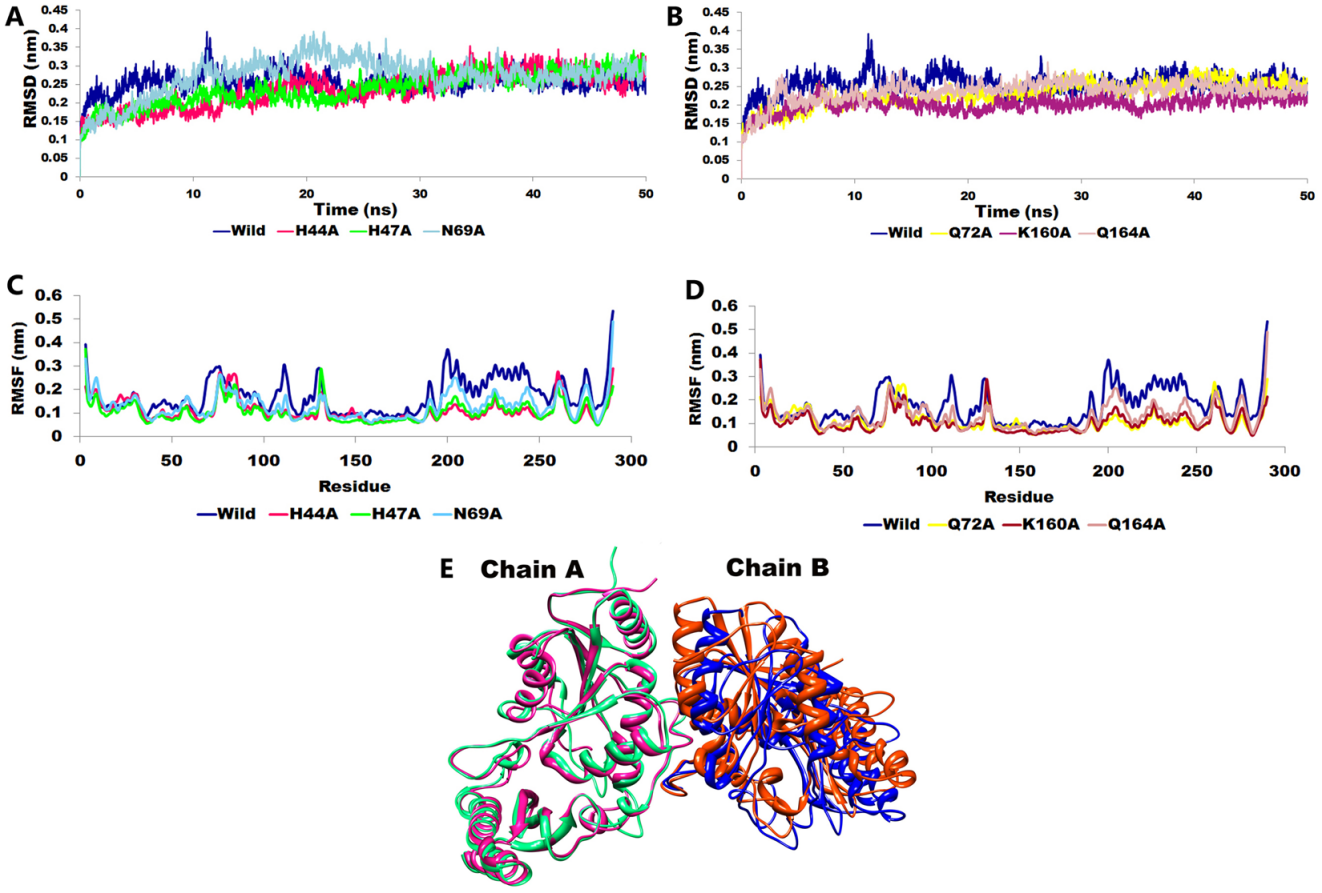
Plot showing the (**A**,**B**) RMSD, (**C,D**) RMSF for wild-type and six alanine mutants and (**E**) Ribbon representation showing superimposition of the pre-MD wild-type PanC homodimer (Chain A: orange, Chain B; Red) and post-MD simulation structure (Chain A: blue, Chain B; green).

RMSD analysis of the protein give insights into its structural conformation during the simulations, providing an indication of the stability of the protein and whether the simulation has equilibrated^14^. The average backbone RMSD for wild-type and six alanine mutants were found to vary between 0.23 to 0.27 nm and remained stable through the entire MD simulations period (Fig. 1A,B). Stable RMSD of the protein till the end of the simulation, suggested that the simulation are perfect for further rigorous analysis. Maximum RMSD fluctuation was seen in mutant N69A between 10–30 ns which indicated that the N69A mutant was undergoing a large conformational change during the simulation. Afterwards, N69A remained stable with average backbone RMSD of ~0.27 nm.

RMSF graph for each residue was calculated and peaks represented local fluctuations along the protein chain during MD simulations. We have observed that both the terminals i.e., N and C-terminals fluctuate more than any other part of protein. Almost all alanine mutants exhibited the same fluctuation for backbone atom in key active site residues. In particular, RMSF value for the active site residue H44, H47, N69, Q72, K160 and Q164 in wild- type was observed to be lower than the alanine mutants (Fig. 1C,D). The complex structures Fig. 1E shows superimposition of the crystallographic structure and the average structure obtained from simulation run with RMSD of 0.9 Å over 290 residues from chain A and B.

### Interaction pattern analysis of PS-ATP complex

In the pantothenate biosynthesis, initially binding of ATP with pantoate in presence of PS resulted in formation of pantoyl adenylate and pyrophosphate. The crystal structure of PS bound with ATP (PDB ID: 2A84) was used for the creation of six alanine mutants and differences in their binding pattern and conformations was studied. Extensive number of hydrogen bonds (H-bonds) (seventeen) play significant role in the stabilization of PS-ATP complex (Table S1). Formation of pantothenate is Mg^2+^ ion dependent reaction and enzyme (PS) has two Mg^2+^ ions; one Mg^2^ showed interaction with ATP whereas another Mg^2+^ ion tends to interact with Asp88, Asp89 and Gln92 residues respectively (Figure S1(B); Table S1)). Furthermore, three alanine mutants, H44A + ATP, H47A + ATP and Q164A + ATP were stabilized by fifteen H-bonds, whereas N69A + ATP, Q72A + ATP, K160A + ATP by thirteen, sixteen and fourteen hydrogen bonds respectively (Table S1). The number of residues involved in the hydrophobic interactions with ATP (eight) remained same for all alanine mutants (Table S1). Mg^2+^ ion interaction pattern was same in all alanine mutants. Two salt bridges were formed by each Lys160 and Arg198 with the phosphate group of ATP molecule also contributed towards complex stabilization in wild-type and all alanine mutants.

### Molecular dynamics simulations of PS-ATP complex

**Figure 2:**
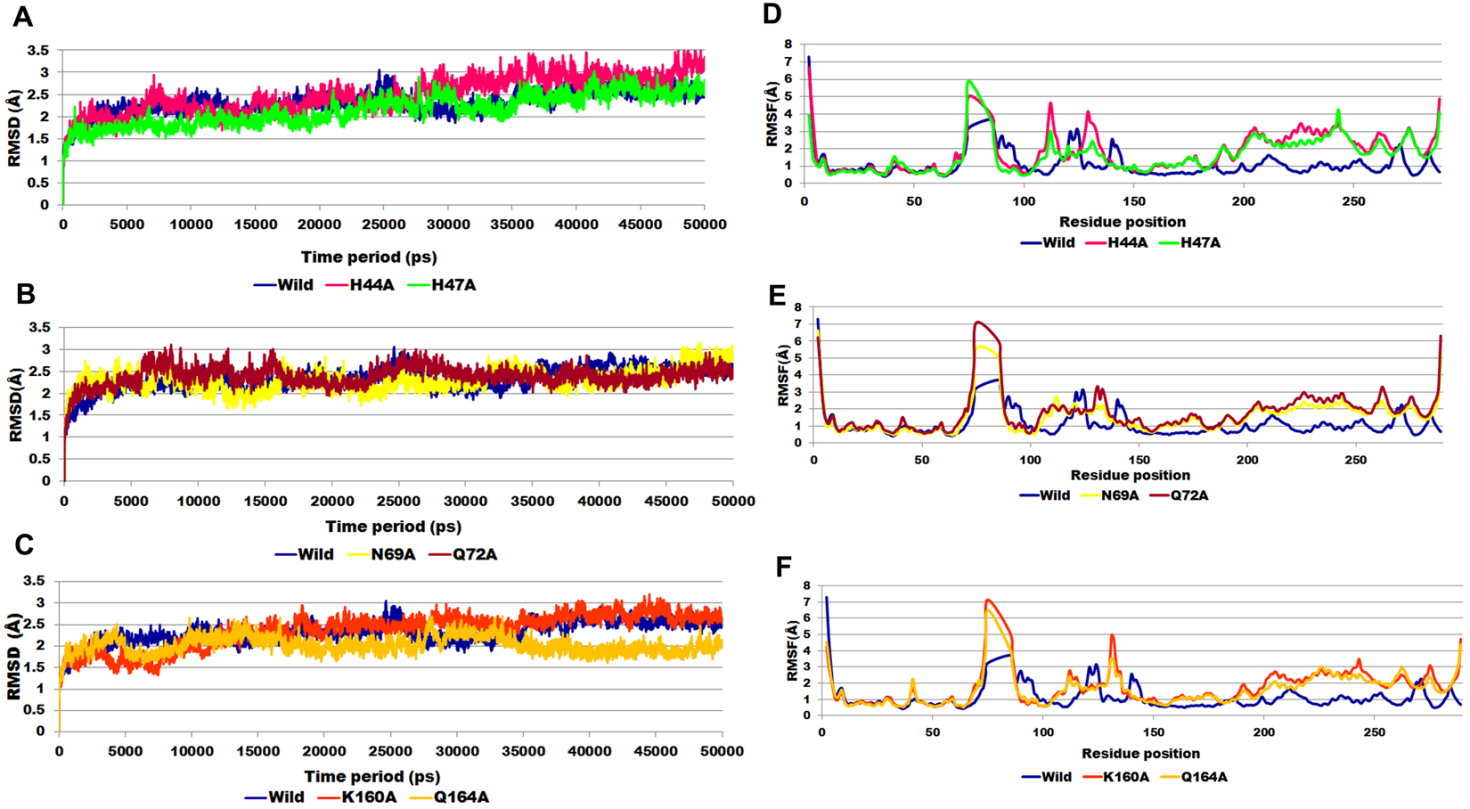
Plot showing backbone RMSD of wild-type and alanine mutant complexes during 50 ns MD simulation with respect to time (**A**) wild-type, H44A and H47A mutants; (**B**) wild-type, N69A and Q72A mutants; (**C**) wild-type, K160A and Q164A mutants. RMSF profile for each residue was depicted for (**D**) wild-type, H44A and H47A mutants; (**E**) wild-type, N69A and Q72A mutants; (**F**) wild-type, K160A and Q164A mutants.

To examine the change in the conformation and stability of the ligand bound complexes, studies demand MD simulations, as it provides valuable clues and details of the key interactions and orientation of the ligand in the final complex structure^15^. Long term MD simulations were performed for each system (wild-type + ATP, H44A + ATP, H47A + ATP, N69A + ATP, Q72A + ATP, K160A + ATP and Q164A + ATP) to study the dynamical behaviour of protein and ligand complex at atomics level. The conformational features of all the systems were analysed using MD trajectories along with the incorporation of various statistical parameters. RMSD for the wild-type and alanine mutants were found to be ≤3.0 Å (except H44A) during the entire simulation run, consequently quantifying conformational changes in the complex. The average backbone RMSD for wild-type, H44A, H47A, N69A, Q72A, K160A and Q164A was found to be ~2.31 Å, 2.49 Å, 2.34 Å, 2.30 Å, 2.39 Å, 2.36 Å and 2.02 Å respectively, suggested that at the time of 50 ns, all the systems achieved the convergence (Fig. 2A–C). Average RMSD value between 1–3 Å are perfectly acceptable whereas value >3 Å showed that protein is certainly undergoing large conformational changes during MD simulations and are pretty meaningless to consider for further analysis^16^. Using stable RMSD time frame, representative structures for each system was extracted for further analysis. The ligand binding poses, energy and interaction fully depends on residual fluctuation (RMSF) values. We also analysed RMSF plot which will depict the ratio of fluctuation in residue level. RMSF of individual amino acid residues was calculated to observe the individual dynamic behaviour of the wild-type and mutant types. The high RMSF value indicates more flexibility whereas the low RMSF value indicates limited movements during simulation in relation to its average position^17^.

RMSF plot for each residue are shown in Fig. 2(D–F), clearly depicting difference in flexibility of for wild-type and alanine mutants residues. It was observed that residues ranged between 60–80 showed highest fluctuation in wild-type and mutants (Fig. 2D–F). All alanine mutants showed similar degree of fluctuation in the residues ranging from 200–250. The highest RMSF reached by wild-type, H44A, H47A, N69A, Q72A, K160A and Q164A was 3.7 Å, 5.0 Å, 5.8 Å, 5.5 Å, 7.0Å, 7.1 Å and 6.5 Å respectively.

The RMSF value of the interactive residues of the active site of the protein was listed in Table 1 indicated that wild-type remained more rigid as compared to the alanine mutants during the simulation time period. It was found that except H44A, which showed low RMSF value at position 44^th^ (Table 1; Fig. 2D), all other mutants exhibited high RMSF value at the alanine substitution position with respect to wild-type, indicating that alanine substitution has induced greater degree of mobility at conserve active site in mutants with higher RMSF value as compared to wild-type. Greater mobility in the active site residues signifies a greater magnitude of flexibility and instability of the enzyme substrate complex^18^.

**Table 1:** RMSF value for the active site residues of wild-type and alanine mutants.

Active site residues	M40	H44	H47	N69	Q72	K160	Q164
**Systems**							
Wild-type	0.88	0.83	0.71	0.81	1.54	0.58	0.49
H44A	1.18	0.80	0.78	1.78	1.56	1.09	0.98
H47A	1.31	1.23	0.86	1.27	1.42	1.15	1.07
N69A	0.75	0.67	0.79	1.51	2.05	0.9	1.00
Q72A	1.09	0.94	0.76	1.62	2.80	1.06	1.15
K160A	1.34	0.89	0.72	1.86	2.49	1.20	1.10
Q164A	1.38	0.81	0.72	1.50	1.87	0.98	1.06

*Values are in Å.

**Figure 3:**
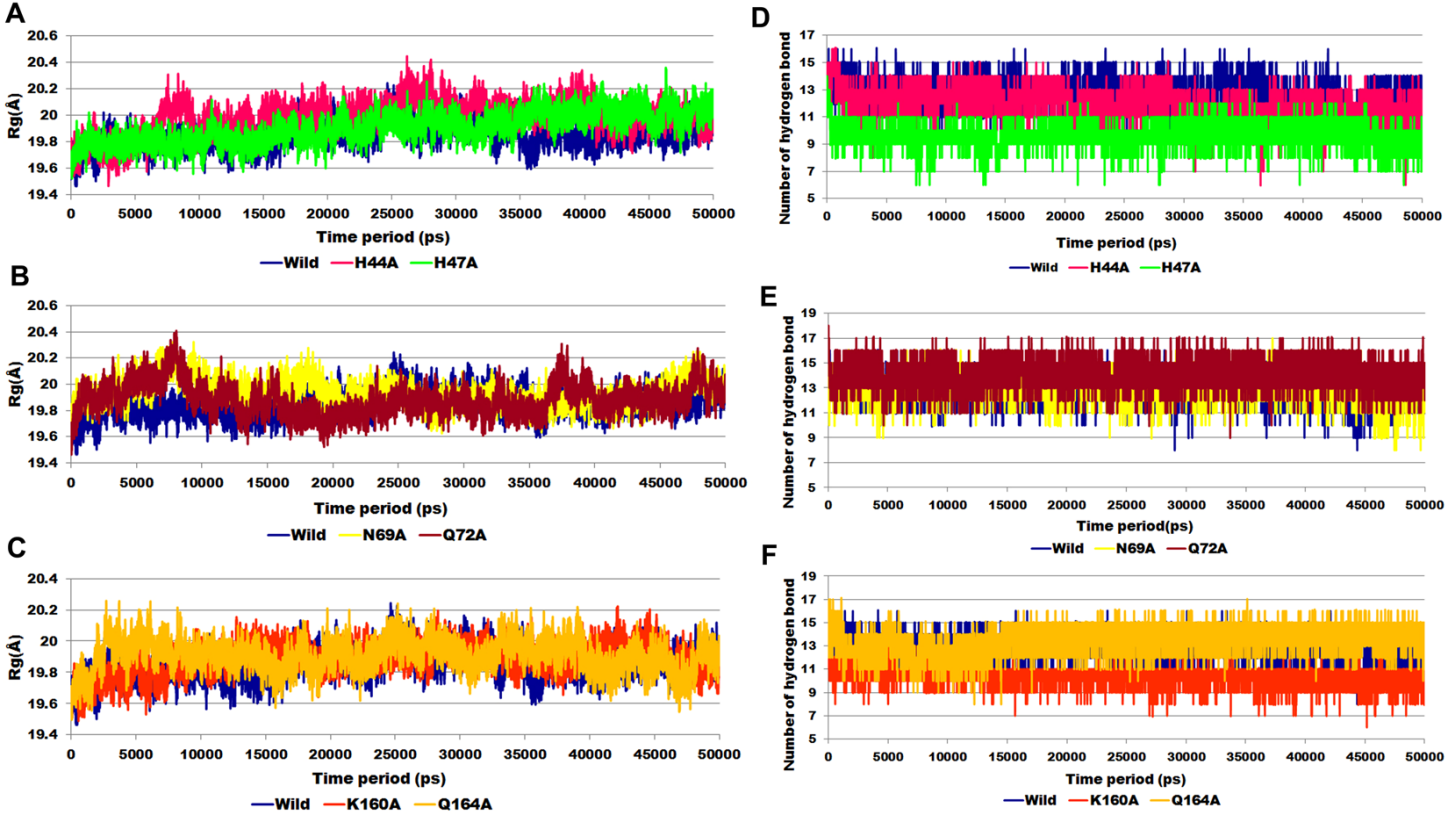
Radius of gyration profile for (**A**) wild-type, H44A and H47A mutants; (**B**) wild-type, N69A and Q72A mutants; (**C**) wild-type, K160A and Q164A mutants. Number of hydrogen bonds computed for (**D**) wild-type, H44A and H47A mutants; (**E**) wild-type, N69A and Q72A mutants; (**F**) wild-type, K160A and Q164A mutants.

Radius of gyration (Rg) is an indicator of structure compactness and overall dimension of protein. It explains how regular secondary structures are compactly packed into 3D structure of protein. If a protein is stably folded, it will likely maintain a relatively steady value of Rg, whereas it will change over time for unfolded proteins^19^. The radius of gyration analysis for the wild-type and alanine mutants showed remarkable differences. We noticed that the wild-type exhibited overall minimum Rg (19.8 Å) value as compared to alanine mutants suggested that wild-type has maintained its compactness during the simulation time period. The graph indicates simultaneous decrease in globularity of alanine mutant as depicted by significant increase in the average Rg score (>19.8 Å). Low value of Rg for the wild-type suggested tight packing of the protein, making the structure relatively stable as compared to the alanine mutants with high Rg value (Fig. 3A–C)^20^.

The hydrogen bonds (H-bonds) formed between the PS and ATP, were identified by measuring the donor-acceptor distances during the MD simulations. The number of H-bonds formed between PS and ATP during the MD simulation in wild-type and mutants is shown in the Fig. 3D–F. With respect to time, wild-type, N69A, Q72A, and Q164A showed average number of H-bonds >12. Whereas, H47A showed lowest number of H-bonds (9) followed by K160A (10) and H44A (11) respectively. Long variation in the RMSD, Rg plot of H44A might be indicative of foremost structural changes which might induce significant decrease in the occupancy of the most H-bonds.

### Calculation of protein binding pockets

For detail investigation of the reason behind the severe difference in the interaction behaviour of protein and structural changes due to alanine substitution, the binding pocket area and volume of the wild-type and mutants was studied. The area and volume of the binding pockets of structural variants of PS was calculated at 0 ns, 10 ns, 20 ns, 30 ns, 40 ns and 50 ns steps of MD simulations using representative structure for all systems^21^. The binding pocket area and volume of the wild-type was found to be 1308.4 Å^2^ and 1889.6 Å^3^ respectively. The representative structures of all alanine mutants except H47A and K160A showed increase in the binding cavity volume and area (Table 2). Significant decrease was observed in H44A with area and volume of 1382.6 Å^2^ and 954.2 Å^3^ respectively. H47A and K160A mutants displayed decrease in the binding pocket volume and area with respect to wild-type, that correlates well with the decrease in the number of H-bonds between PS an ATP during MD simulation time period. Decrease in enzyme binding pocket in alanine mutants does not allow substrate (ATP) to fit well into the binding cavity and form reaction intermediates^22–24^. The slight increase in the binding pocket area (N69, Q72, and Q164) triggers larger area of contact between enzyme and substrate with more effective hydrogen bonding (as shown in Figure S2(A–G)).

**Table 2:** Binding cavity size analysis for wild-type PS and alanine mutants.

**Time**	**0 ns**	**10 ns**	**20 ns**	**30 ns**	**40 ns**	**50 ns**	**Average Structure**
**Wild-type**							
Volume (Å3)	1717	1461.7	1665	1844.3	1870.3	1979.9	1889.6
Area (Å2)	1203.2	1000.4	1047.1	1370.6	1452.5	1108.3	1308.4
**H44A**							
Volume (Å3)	1848.2	1153.5	1837.2	1721.4	1907.5	2297.2	1382.6
Area (Å2)	1318.7	1004	1295.2	1225	1202.5	1423.5	954.2
**H47A**							
Volume (Å3)	1950.3	1639	1368.3	1762	1674	2015.1	1723
Area (Å2)	1316.8	1171	1009	1343.2	1268.2	1409.9	1218
**N69A**							
Volume (Å3)	2025.5	2489.4	2830.4	2497	2285.6	2990.1	2149.8
Area (Å2)	1183.3	1619.6	1929.3	1788.7	1729.4	1912.6	1598.7
**Q72A**							
Volume (Å3)	1923.2	2131	2541.5	2628.4	2388.5	2592	2145.5
Area (Å2)	1639.7	1309	1691.4	1832.8	1516.4	1670.9	1353.2
**K160A**							
Volume (Å3)	1742.6	2138.4	2527.9	2883.3	1827.4	1824.9	1750.6
Area (Å2)	1245.3	1370.3	1518.4	1568.3	1221.2	1107.3	1211.8
**Q164A**							
Volume (Å3)	1920.4	2015.6	2483.4	2529.7	2207.3	2493.7	2061.9
Area (Å2)	1168.4	1448.6	1538.2	1419.3	1559.5	1802	1335

### Effect of mutation on binding free energy analysis

MM-GBSA method was used to predict relative changes caused in binding free energy due to alanine substitution in the active site residues^25^. Prime MM-GBSA takes less computational power and reported to produce results with good accuracy. It generates a lot of energy properties including energies for the ligand, receptor, and complex structures as well as energy differences relating to strain and binding. We have reported total binding energy (deltaG value) of the complex, contributed from various energy terms before and after MD simulations. The binding free energy value of for the wild-type was found to be −29.71 kcal/mol whereas H44A, N69A, Q72A and Q164A mutants exhibited slightly higher binding energies (ranged between −29.97 to −32.00 kcal/mol) than the wild-type in the pre MD simulated complexes (Table 3). On the contrary lowest binding free energy was exhibited by H47A and K160A mutants as compared to the wild-type, implied that alanine substitution at 47^th^ and 160^th^ position has caused drastic effect on the binding affinity of the PS. Additionally, MM-GBSA calculation was also computed for MD simulated complexes to explore the stability and conformational changes in the complexes. MM-GBSA results for simulated complexes showed that wild-type exhibited highest binding free energy of −26.61 kcal/mol as compared to all alanine mutants except Q72A (−27.28 kcal/mol), suggesting high affinity for the ATP. Thus MM-GBSA analysis for both the pre- and post-MD structures showed similar profiles, making us conclude that alanine mutations at H47 and K160 active site residues have most severe impact on stability and catalytic activity of the enzyme (Table 3). Moreover, previous studied have investigated the free energy change of ATP in tightly bound, bound and empty stages and it was found that ATP in tight bound state has large negative free energy^26,27^. Reduction in binding affinity in the alanine mutation may be result of several factors, including the conformation change due to mutation which made binding of ATP less pronounced with PS. Whereas, increase in the binding free energy in N69A, Q72A and Q164A, suggested that alanine substitution severely disturbs protein-ligand interaction and thereby increasing or decreasing the binding free energy^28^. Our results strongly correlated with the experimental data from isothermal titration microcalorimetry (ITC) analysis which showed that as compared to the wild-type, the association constant (Ka) for ATP to H47A and K160A decreased by ~10 and 50 fold while no significant difference was observed for Q72A, Q164A and N69A mutants (<~2 fold) (Table 3). The enthalpy (∆H) for ATP binding to PS ranged from −17.3 to −13.0 kcal/mol for wild-type, Q72A, Q164 and N69A mutants, whereas it was found to be −7.2 and −8.4 kcal/mol for H47A and K160A mutants (Table 3). In addition, the −T∆S values for the binding of ATP with Q72A, Q164 and N69A were highly negative but the values for H47A and K160A were small. Similarly, the difference between ∆G values (free energy) between wild-type and Q72A, Q164 and N69A were not very significant. On the other hand mutants H47A and K160A were found having prominent changes in binding energy values with respect to wild-type (difference of −1.3 and −2.3 kcal/mol respectively with respect to wild-type)^6^. Due to the precipitation of H44A mutant during experimentation, its thermodynamic data could not obtained. Therefore, it was concluded that alanine mutation at H47 and K160 active site residues have most severe impact on stability and catalytic activity of the enzyme.

**Table 3:** Predicted experimental values and comparative binding free energies for wild-type and alanine mutants.

Systems	Isothermal titration microcalorimetry results				MM-GBSA	
	Ka(x10^4^ M^−1^)	−∆G (kcal mol^−1^)	−∆H (kcal mol^−1^)	−T∆S (kcal mol^−1^)	Pre-MD (kcal mol^−1^)	Post-MD (kcal mol^−1^)
Wild-type	22.4 ± 1.6	7.3	14.2 ± 0.2	6.9	−29.71	−26.61
H44A	—	—	—	—	−29.97	−21.70
H47A	2.46 ± 0.11	6.0	7.2 ± 0.2	1.2	−28.56	−20.76
N69A	9.1 ± 0.4	6.8	13.0 ± 0.23	6.1	−30.39	−22.33
Q72A	13.2 ± 0.29	7.0	13.1 ± 0.46	6.2	−32.00	−27.28
K160A	0.45 ± 0.02	5.0	8.4 ± 0.3	3.4	−25.24	−17.93
Q164A	13.2 ± 0.29	7.0	17.3 ± 0.1	10.3	−30.49	−26.47

“—”: not predicted.

### Analysis of Interaction pattern after MD simulations

**Figure 4:**
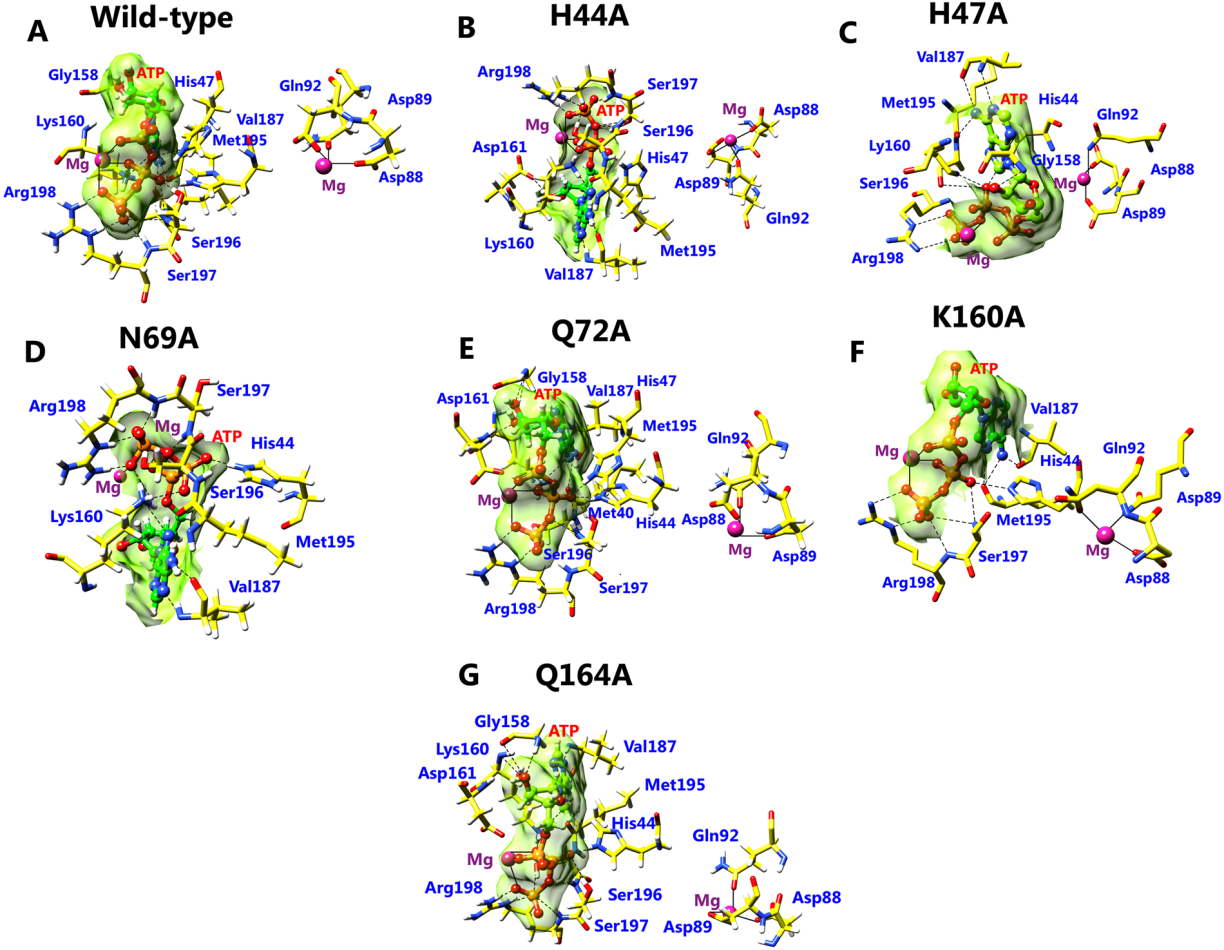
Illustration of bonding interaction pattern in the (**A**) wild-type, (**B**) H44A (**C**) H47A (**D**) N69A (**E**) Q72A (**F**) K160A (**G**) Q164A after 50 ns MD simulations. Interaction of Mg2+ ion with ATP and PS residues is represented as “–” and H-bond as “------” respectively.

Interaction between PS and ATP in the wild-type and mutant were investigated after MD simulations. During the course of MD simulations, wild-type complex was stabilized by sixteen hydrogen bond and five residues were involved in hydrophobic interactions (Fig. 4A; Table 4). Similar salt bridge interactions were retained in the post simulated structure demonstrated that enzyme and substrate have high affinity and interact strongly. H44A, N69A, Q72A and Q164 mutant complexes stabilized the complex formation with ATP with sixteen (highest), fifteen, thirteen and twelve number of H-bonds (Fig. 4B,D,E,G; Table 4). In addition, H44A, H47A and Q72A complexes were also stabilized by non-covalent interactions such as pi-pi interaction was formed by His44 (H44A complex) and His47 (H44A and Q72A complexes) with adenine base of ATP. Interestingly, H47A and K160A complexes showed loss of hydrogen bonding in the simulated complex, hence reducing the binding affinity between PS and ATP (Fig. 4C,F; Table 4).

**Table 4:** Interaction profile between PS and ATP in wild-type and alanine mutants after MD simulations.

Systems	No. of hydrogen bond	Participating residues in hydrogen bond with their bond length (Å)	Participating residues in hydrophobic bonding
Wild-type	16	Arg44(2.7 Å), Arg47(2.7 Å, 2.9 Å), Gly158(2.7 Å,), Lys160(2.5 Å, 2.9 Å), Val187(3.0 Å, 3.0 Å), Met195(2.9 Å), Ser196(2.7 Å), Ser197(3.1 Å, 2.8 Å, 2.6 Å), Arg198(2.8 Å, 2.7 Å, 2.5 Å)	Gly46, Leu50, Phe157, Pro185, Thr186
H44A	13	Arg47(2.6 Å), Lys160(2.5 Å, 3.0 Å), Asp161(2.5 Å), Val187(2.9 Å, 2.9 Å), Met195(2.9 Å), Ser196(2.7 Å), Ser197(2.9 Å, 3.0 Å), Arg198(3.2 Å, 2.6 Å, 2.5 Å)	Gly46, Phe157, Gly158, Pro185, Thr186
H47A	9	Arg44(2.8 Å), Gly158(2.9 Å,), Lys160(2.7 Å), Val187(2.9 Å, 3.1 Å), Met195(2.9 Å), Ser196(2.6 Å), Arg198(2.6 Å, 2.5 Å)	Gly46, His47, Leu50, Phe157, Pro185, Thr186
N69A	12	Arg44(2.6 Å), Lys160(2.9 Å, 2.6 Å, 2.9 Å), Val187(3.0 Å, 3.0 Å), Met195(3.0 Å), Ser196(2.6 Å), Ser197(2.7 Å), Arg198(2.8 Å, 2.6 Å, 2.5 Å)	Met40, Gly46, His47, Leu50, Phe157, Gly158, Thr186
Q72A	16	Arg44(2.7 Å), Arg47(2.6 Å), Gly158(3.0 Å), Lys160(2.5 Å, 2.9 Å), Asp161(2.9 Å),Val187(2.9 Å, 2.8 Å), Met195(3.0 Å), Ser196(2.6 Å), Ser197(2.9 Å, 2.9 Å, 3.1 Å), Arg198(2.7 Å, 2.7 Å, 3.1 Å)	His44, Leu50, Phe157, Pro185, Thr186
K160A	9	Arg44(2.6 Å), Val187(2.9 Å), Met195(2.8 Å, 3.3 Å), Ser197(3.2 Å, 2.8 Å), Arg198(2.9 Å, 2.9 Å, 2.9 Å)	Gly46, His47, Leu50, Phe157, Ala160, Pro185, Thr186
Q164A	15	Arg44(2.6 Å), Gly158(2.9 Å, 3.3 Å), Lys160(2.5 Å, 2.8 Å), Asp161(3.2 Å) Val187(2.9 Å, 2.7 Å), Met195(3.0 Å), Ser196(2.6 Å), Ser197(2.8 Å, 3.1 Å), Arg198(2.5 Å, 2.6 Å, 3.0 Å)	Met40, His47, Leu50, Phe157, Pro185, Thr186

The interaction pattern results are in very well agreement with the binding pocket analysis, and MD trajectory outcome for the wild-type and mutants. The residues involved in the hydrophobic interactions in all systems are mentioned in Table 4. Salt bridge and Mg^2+^ ion interactions were retained in the post simulated structure of wild-type and alanine mutant complexes. The results indicated that alanine substitution has induced decrease in the occupancy of hydrogen bonds (except Q72A and Q164A mutants), resulting in a loss of enzyme activity and reduce affinity for ATP. Overall, the interaction pattern before and after the MD simulations implies consistency in the stability of wild-type PS in comparison to the alanine mutants, which is necessary for the formation and stability of pantoyl adenylate intermediate in the pantothenate biosynthesis pathway.

### Effect of mutation on secondary structure

Time dependent changes in the secondary structure elements were computed for the wild and mutant protein complex. Secondary structure elements helix (α) and beta (β) sheets are rigid in nature whereas coli, turns and loops are flexible. The structural changes were computed for 50 ns time period. Increase in α-helix was observed in all alanine mutants except K160A whereas Q164A accounted highest percentage for α-helix and Q72A remained unchanged as compared to the wild-type. When compared to wild-type, H47A, Q72A, K160A and Q164A mutants exhibited increment in β-sheet. Decrease in percentage of 3_10_-heix was observed in H44A, H47A and K160A mutants whereas it was found to increase in Q72A and lost in Q164A mutant with respect to wild-type (Table S2; Figure S3). In term of flexible secondary structure, coil was found to increase in H44A and Q72A whereas K160A showed increment in turns as compared to wild-type. In addition, all alanine mutants showed increase in percentage of isolated β-bridge. Secondary structure for each conserve active site residue was calculated and reported in Table S2.We observed conversion from helix to turn at 47^th^ position and turn to 3_10_helix at 160^th^ position in H47A and K160A mutants when compared to wild-type (Table 5). Notably, formation and loss of secondary structure during simulation time period was observed in all mutants as compared to wild-type, thus concluding that mutation leads to change in the structural flexibility and protein conformation^29^.

**Table 5:** Secondary structure analysis for the conserve active residues in wild-type and alanine mutants.

Active site residues	M40	H44	H47	N69	Q72	K160	Q164
**Systems**							
Wild-type	Coil	Coil	Helix	Coil	Turn	Turn	Helix
H44A	Coil	Coil	Helix	Coil	Coil	Turn	Helix
H47A	Strand	Turn	Turn	Coil	Turn	Turn	Helix
N69A	Coil	Coil	Helix	Coil	Turn	Turn	Helix
Q72A	Strand	Coil	Helix	Coil	Turn	Turn	Helix
K160A	Strand	Turn	Helix	Coil	Turn	3_10_helix	Helix
Q164A	Coil	Coil	Helix	Coil	Turn	Turn	Helix

### Residue network analysis

Protein function and its structural stability depend on the inter-residue interaction network. The residue interaction analysis (RIN) is representations of protein network were amino acid residues are nodes and arcs are interaction between them. RIN has been extensively used to study the effect of mutation on protein interaction network^30^. The average structures of wild-type and mutants were used for generation of residue interaction network. The interaction formed by the active site residues was studied in wild-type and alanine mutants. The comparative residue network analysis displayed maximum interaction loss was observed in H44A, H47A, N69A and K160A mutants whereas Q72A and Q164A mutants retained most of the residue interactions as compared to the wild-type (Table 6; Figure S4). Investigation of residue network indicated that alanine substitution destabilizes the interaction of active site residue resulting in distorted geometry of active site. In addition, ATP network with the protein residue (distance < 6.0 Å) was also studied, and results were in agreement with the interaction analysis performed before and after MD simulations of wild-type and mutants (Table S3; Figure S5).

**Table 6:** Interaction of active site residues in wild-type and alanine mutated complexes.

Interactions	Wild-type	H44A	H47A	N69A	Q72A	K160A	Q164A
44–47	H. bond, VDW	H. bond, VDW	H. bond, VDW	H. bond, VDW	H. bond, VDW	H. bond, VDW	H. bond, VDW
44–48	H. bond	H. bond, VDW	H. bond,	H. bond, VDW	H. bond, VDW	H. bond,	H. bond,
44–195	VDW	—	VDW	—	VDW	VDW	VDW
44–196	—	—	—	—	VDW	—	VDW
44–197	H. bond	—	VDW	—	VDW	—	—
47–50	H. bond	H. bond	—	H. bond	H. bond	H. bond	H. bond
47–197	—	—	—	—	VDW	—	—
69–139	—	—	—	—	—	—	VDW
72–114	—	—	—	—	—	—	VDW
72–117	—	—	VDW	—	—	—	—
72–134	—	—	—	—	VDW	VDW	—
72–135	VDW	VDW	—	—	VDW	—	H. bond
72–139	VDW	—	—	—	—	—	VDW
160–186	—	—	VDW	VDW	—	—	—
160–196	H. bond	H. bond	—	H. bond	H. bond	—	H. bond
160–194	—	—	—	—	—	—	VDW
160–195	H. bond	H. bond	—	H. bond	H. bond	—	H. bond
160–198	—	—	—	VDW	—	—	—
160–282	—	—	—	—	H. bond	—	H. bond
164–157	VDW	—	—	—	—	—	—
164–167	—	VDW	H. bond	—	H. bond	H. bond	—
164–168	H. bond	H. bond, VDW	H. bond, VDW	H. bond	H. bond, VDW	H. bond	—

^*^“VDW”: Van der Waals, “—”: not present.

### Principal component analysis and free energy landscape

The collective motion of the wild-type and mutant proteins was studied from the MD trajectories using principal component analysis (PCA). PCA method is based on construction of diagonal covariance matrix from Cα atom of the protein that captures strenuous motion of the atom through eigenvectors and eigenvalues^31^. Eigenvectors elucidate the overall direction of motion of the atoms whereas eigenvalues represent the atomic contribution of motion. To better understand the structure and conformational changes, MD trajectories of the wild-type and mutant structures in ligand bound form were examined with the principal component^32^.

**Figure 5:**
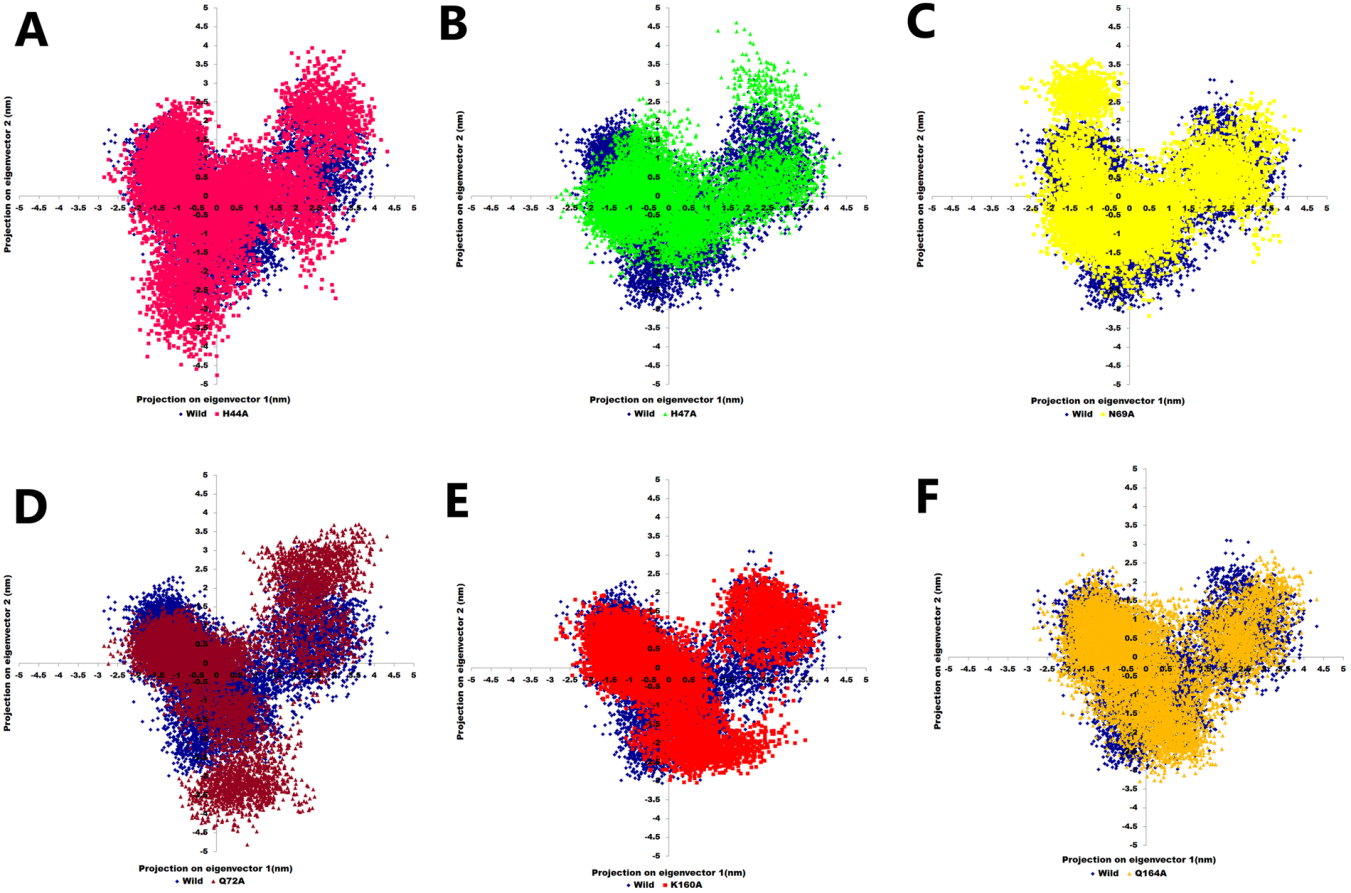
Projection of protein atoms in phase space along the first two principal eigenvectors (**A**) wild-type and H44A mutant (**B**) wild-type and H47A mutant (**C**) wild-type and N69A mutant (**D**) wild-type and Q72A mutant (**E**) wild-type and K160A mutant (**F**) wild-type and Q164A mutant.

The corresponding eigenvalues indicated the dynamic behavior and degree of fluctuation of wild-type and mutant proteins. The trace value for wild-type, H44A, H47A, N69A, Q72A, K160A and Q164A complexes was found to be 8.4 nm^2^, 14.4 nm^2^, 9.9 nm^2^, 11.3 nm^2^, 14.7 nm^2^, 13.1 nm^2^ and 12.2 nm^2^ respectively. All the alanine mutants showed high values indicating escalation in collective motion of the protein as compared to wild-type variant during the simulation. As a result of greater flexibility, conformational space covered by mutant PS complexes was wider than the wild-type complex^33^ (Fig. 5A–F). Thus, from above results it was concluded that wild-type complex was more stable than the mutant protein complexes.

The cosine content (ci) of the principal component (pi) of covariance matrix (C) is an absolute convergence measure that was calculated using covariance analysis and ranges between 0 (no cosine) and 1 (perfect cosine):

2 $$\,{c}_{i}=\frac{2}{T}{(\int \cos (i\pi t){p}_{i}(t)dt)}^{2}{(\int {p}_{i}^{2}(t)dt)}^{-1}$$

where T is the total simulation time.

**Figure 6:**
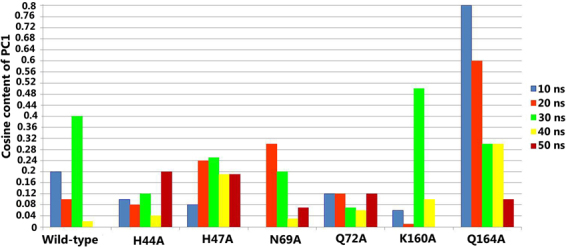
Cosine content of the first principal component analysis for MD simulation trajectories of wild-type and alanine mutants.

The examination of first eigenvector for cosine contribution gives enough idea about the protein behavior^34^. Cosine content close to 1 represents random motion in the protein hence cannot be considered for free energy landscape analysis. It was reported that cosine content near to 0.2 and 0.5 produce reliable free minimum energy clusters^35^. Therefore we calculated cosine content for the first eigenvector for wild-type and mutants on 50 ns MD trajectory. We observed that cosine contents for wild-type, H44A, H47A, N69A, Q72A, K160A and Q164A was close to zero (0) at 50 ns, confirming coordinated motion within the protection due to convergence of simulation at the 50 ns time period and hence energy landscape analysis was carried out (Fig. 6).

**Figure 7:**
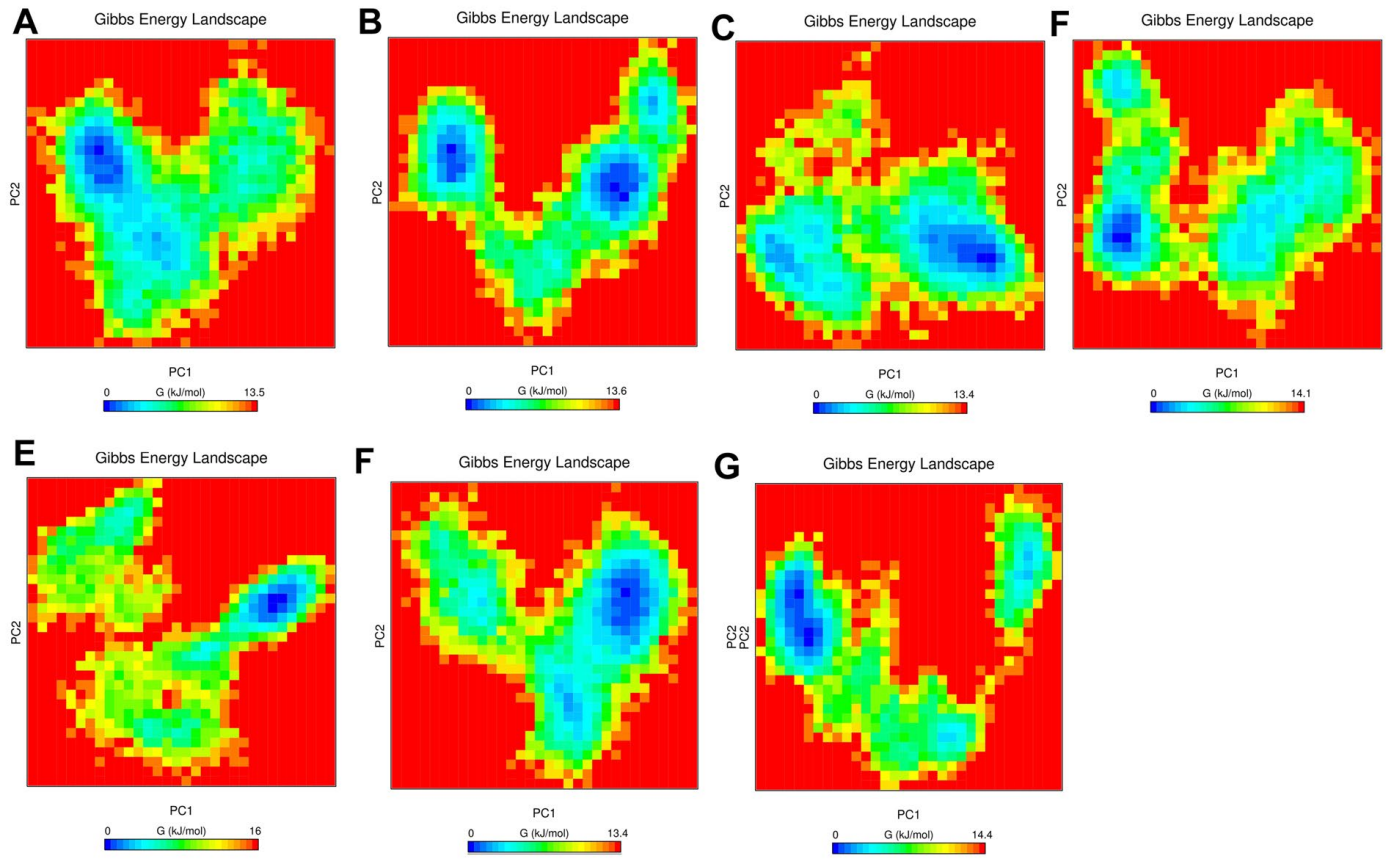
Free energy landscape of the first two principal components for (**A**) wild-type (**B**) H44A (**C**) H47A (**D**) N69A (**E**) Q72A (F) K160A (**G**) Q164A alanine mutants.

To visualize the energy minima of the landscape of ATP bound wild-type and mutant PS (Fig. 7A–G), we studied the FEL against first two principal components PC1 and PC2 which revealed ∆G value 0 to 13.5, 13.6, 13.4, 14.1, 16, 14.4 and 13.4 kJ/mol for H44A, H47A, N69A, Q72A, K160A and Q164A mutants respectively. The size and shape of the minimal energy area (in blue) reveals the stability of a complex^36,37^. A single centralized and concentrated energy depicted in wild-type, Q72A and Q164A indicated minimum structural changes as compared to H44A, H47A, N69A and K160A which showed two or several minima.

## Discussion

The biosynthetic pathway of pantothenate is essential for the growth Mtb and comprised four step reaction catalyzed by *panB, panC, panD* and *panE* genes^38^. The final step in the formation of pantothenate is catalyzed by *panC* gene via ATP dependent condensation of D-pantoate and β-alanine. Therefore, PS is very good drug target for developing new drugs against TB^13^. Many inhibitors against PS has been reported which include pyrazolo[4,3-c]pyridine carboxamides derivatives^39^, 3-Biphenyl-4-Cyanopyrrole-2-Carboxylic Acids derivatives^40^, and 2,6-disubstituted 4,5,6,7-tetrahydrothieno[2,3-c]pyridine-3-carboxamide derivatives^41^. There is a dire necessity for investigating the mechanism to reduce the catalytic activity of enzymes which are crucial targets against tuberculosis. Targeting an important enzyme like Pantothenate Synthetase could be a promising approach for developing much-needed new inhibitors aimed at treating tuberculosis infection.

The sequence alignment of PS from *Escherichia coli* and Mtb revealed high degree of the active site of residues^8^. In order to evaluate the role of conserved active site residue Zheng *et al*.^6^ generated six alanine mutants at position H44, H47, N69, Q72, K160 and Q164 and observed the catalytic effect on both the overall reaction and isolated step of adenylation and amide formation. It was found the substitution of alanine with any of these residue leads to significant decrease in the enzyme activity. Alanine mutagenesis studies has been widely studied in various enzyme such as elastase and glucokinase from *Pseudomonas aeruginosa*^42^ and human^43^ to emphasize the role of functionally important residues. In addition, computational studies was performed to predict the effect of alanine mutagenesis in G-protein coupled receptors (GPCR) using MD simulations^44^, which proved to be critical approach for the detection of functionally important active site residues. To gain structural insight into dynamic modulation of PS and interpret the effect of alanine mutation on the conserved residues, we performed extensive computational analysis such as molecular dynamics simulations, PCA and FEL analysis. MD simulations trajectories analysis (RMSD, RMSF, Rg and Hydrogen bond) of the wild-type and mutant complexes, indicated stronger and energetically more favorable interactions in the wild-type, Q72A and Q164A and less favorable in the H47A and K160A mutants whilst, stability profile of H44A and Q69A remained unclear. Similarly another study demonstrated that alanine mutagenesis at residues Q292, F286, K321 and R627 in RecG enzyme eliminated its unwinding activity in Mtb^45^, indicating that these residue are essential for RecG activity.

Furthermore, MM-GBSA results also indicated lowest binding free energy for H47A and K160A mutants and highest for wild-type, Q72A and Q164A with prominent number of hydrogen bonds in pre- and post-MD simulation complexes. Results obtained from ITC also showed less −T∆S and ∆G value for H47A and K160A mutants as compared to wild-type and other alanine mutants^6^. Therefore, the binding energy results were in agreement with the ITC results of PS affinity for ATP. Decline of rigid structure (helices) and residue network interaction was also observed in H44A, H47A, and K160A mutants as compared to wild-type, thus concluding that mutation leads to change in protein conformation and destabilization of the mutant complexes. The PCA and FEL analysis also showed conformational changes in the alanine mutants thus inhibiting the formation of pantoyl intermediate (unable to show high affinity for ATP). The results suggested that these conserved residues are important for stability, catalytic efficiency and unfolding cooperativity of PS enzyme. The results also revealed that substituting conserved active site residue with alanine at 47^th^ and 160^th^ position could be sustainable approach for the for designing PS variants with decreased catalytic activity. This study also promotes the use of computational approach for identifying active inhibitors as enzyme substrate which will destabilize the reaction intermediates in PS catalyzed reaction in rapid, time and cost-effective way.

## Material and Methods

### Protein preparation

Experimentally determined structures of MtbPS in unbound [PDB ID: 1MOP] and bound with ATP (PDB ID: 2A84) were retrieved from RCSB Protein Data Bank (PDB) for our study^4^. In order to determine the functional role of the conserved residues we created six alanine mutants of MtbPS by substituting H44, H47, N69, Q72, K160, and Q164 residues in the coordinate file of MtbPS (1MOP and 2A84) through Swiss Pdb Viewer^46^. The systems were prepared via Schrodinger’s protein preparation wizard^47–49^.

### Molecular dynamics studies

MD simulations of unbound PS, wild-type + ATP, H44A + ATP, H47A + ATP, N69A + ATP, Q72A + ATP, K160A + ATP and Q164A + ATP were carried out Desmond Molecular dynamics system in using similar methodology as elaborated in our previous work^50,51^. All the systems were solvated in cubic water box with the distance of 10 Å between protein and edge of the simulation box using Simple Point Charge (SPC) water model^52^. System was neutralized by adding ions (Na^+^ and Cl^−^) followed by energy minimization. The system was equilibrated using Desmond’s default parameters. Finally, the individual systems were subjected to production run for 50 ns at 300 K and 1 bar pressure.

### MD trajectory analysis

The most important analysis which reflects scientific goal MD simulations was calculation of root mean square deviation (RMSD), the radius of gyration (Rg), root mean square fluctuation (RMSF) and hydrogen bond (H-bond) using inbuilt python script in Schrodinger’s Desmond package. The images were prepared using UCSF Chimera and PyMol. Representative simulated structures for each system was extracted using python script. The interpretation of the hydrogen and hydrophobic interactions was carried out using Ligplot+ (https://www.ebi.ac.uk/thornton-srv/software/LigPlus/). Secondary structure was analyzed for all systems (wild and alanine mutants) using structure and MD simulated trajectory file in VMD program^53^. Binding pocket calculation was performed for wild-type and alanine mutants after the removal of ATP from the PS-ATP complex (PDB ID: 2a84) at the interval of 10 ns using CASTp server^21^.

### Binding free energy calculation

Prime MM/GBSA module of Schrodinger suite^54^ was used to determine binding strength for ATP-PS complexes using molecular mechanics combined with generalized Born (MM/GBSA) approach^55,56^. The binding free energy was calculated using following equation:

3 $${E}_{\mbox{--}}\mathrm{binding}={\rm{E}}({\rm{complex}})-[{\rm{E}}({\rm{receptor}})+{\rm{E}}({\rm{ligand}})]$$

### Residue interaction network analysis

The representative structure for each system (wild-type and alanine mutants) was submitted to RING server^30^. The network was visualized and analyzed using RINalyzer and StructureViz module of Cytoscapev3.5.1^57^. In protein-ligand 3D graph, nodes represented residues and edges symbolized different type of interactions such as van der Waals, ionic interactions and hydrogen bonds.

### Principal component analysis (PCA) and free energy landscape analysis (FEL)

The Desmond simulation trajectories were converted to gromacs trajectories through VMD program^53^. PCA was done with least squares fit to the average structure based on the protein backbone coordinates using gmx-covar and gmx-anaeig gromacs inbuilt tool^58^. Cosine content for the first principal component analysis was computed using gmx-analyze. The FEL created by gmx-sham is based on the probability of given combinations of data points, which are then converted to a free energy value by simple relationships.

## Electronic supplementary material

Supplementary Information
