# Supplementary material for: Alanine mutation of the catalytic sites of Pantothenate Synthetase causes distinct conformational changes in the ATP binding region
Source: Sci Rep. 2018 Jan 17;8:903. doi: 10.1038/s41598-017-19075-2 (PMC5772511; doi:10.1038/s41598-017-19075-2)
Supplement: Supplementary file 1 — Supplementary Information [file 41598_2017_19075_MOESM1_ESM.docx]

**Alanine mutation of the catalytic sites of Pantothenate Synthetase causes distinct conformational changes in the ATP binding region**

Bharati Pandey^1^, Sonam Grover^2^, Sukriti Goyal^4^, Anchala Kumari^5^, Aditi Singh^5^, Salma Jamal^4^, Jagdeep Kaur^1^ and Abhinav Grover^3^*

^1^Department of Biotechnology, Panjab University, Chandigarh, India - 160014

^2^Kusuma School of Biological Sciences, Indian Institute of Technology Delhi, New Delhi, India - 110016

^3^School of Biotechnology, Jawaharlal Nehru University, New Delhi, India - 110067

^4^Department of Bioscience and Biotechnology, Banasthali University, Tonk, Rajasthan, India - 304022

^5^Department of Biotechnology, TERI University, VasantKunj, New Delhi, India - 110070

^*^Corresponding author

**Abhinav Grover**,

School of Biotechnology, Jawaharlal Nehru University, New Delhi, India – 110067

Tel: +91-8130738032; Fax: +91-11-26742040;

Email: [abhinavgr@gmail.com](mailto:abhinavgr@gmail.com), agrover@jnu.ac.in


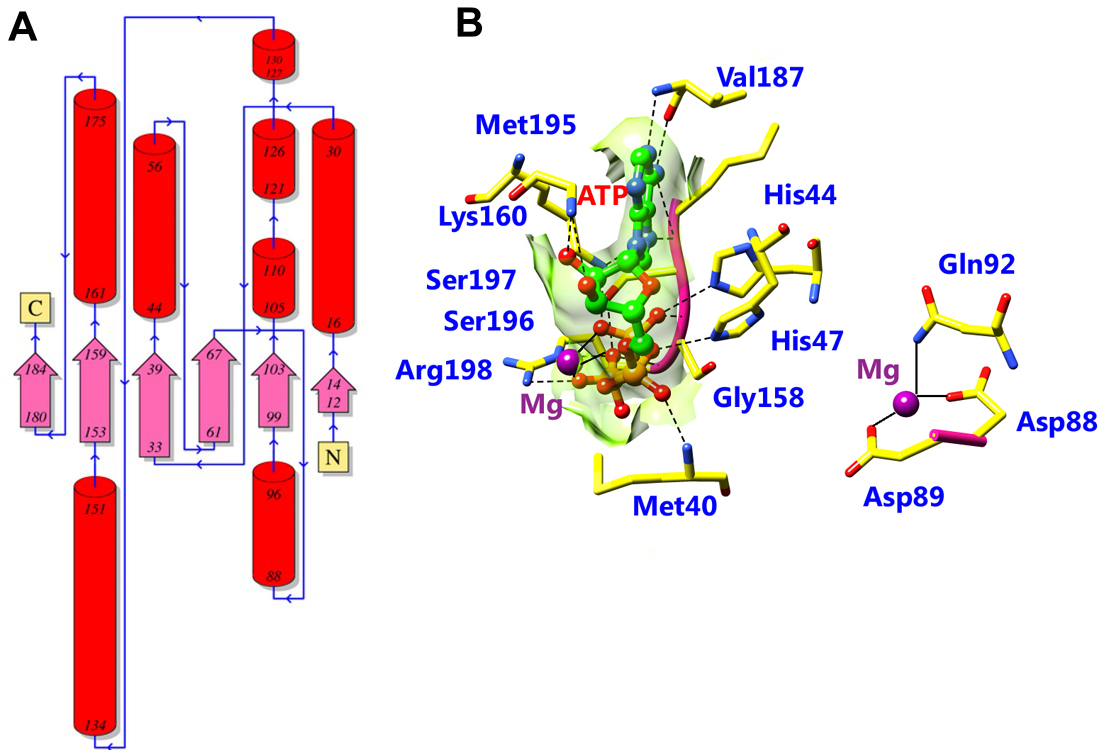


**Figure S1:** (A) Secondary structure representation in MtbPS and (B) Hydrogen bond interaction pattern between PS and ATP. Mg ions also showed interaction with ATP and amino acid residues.


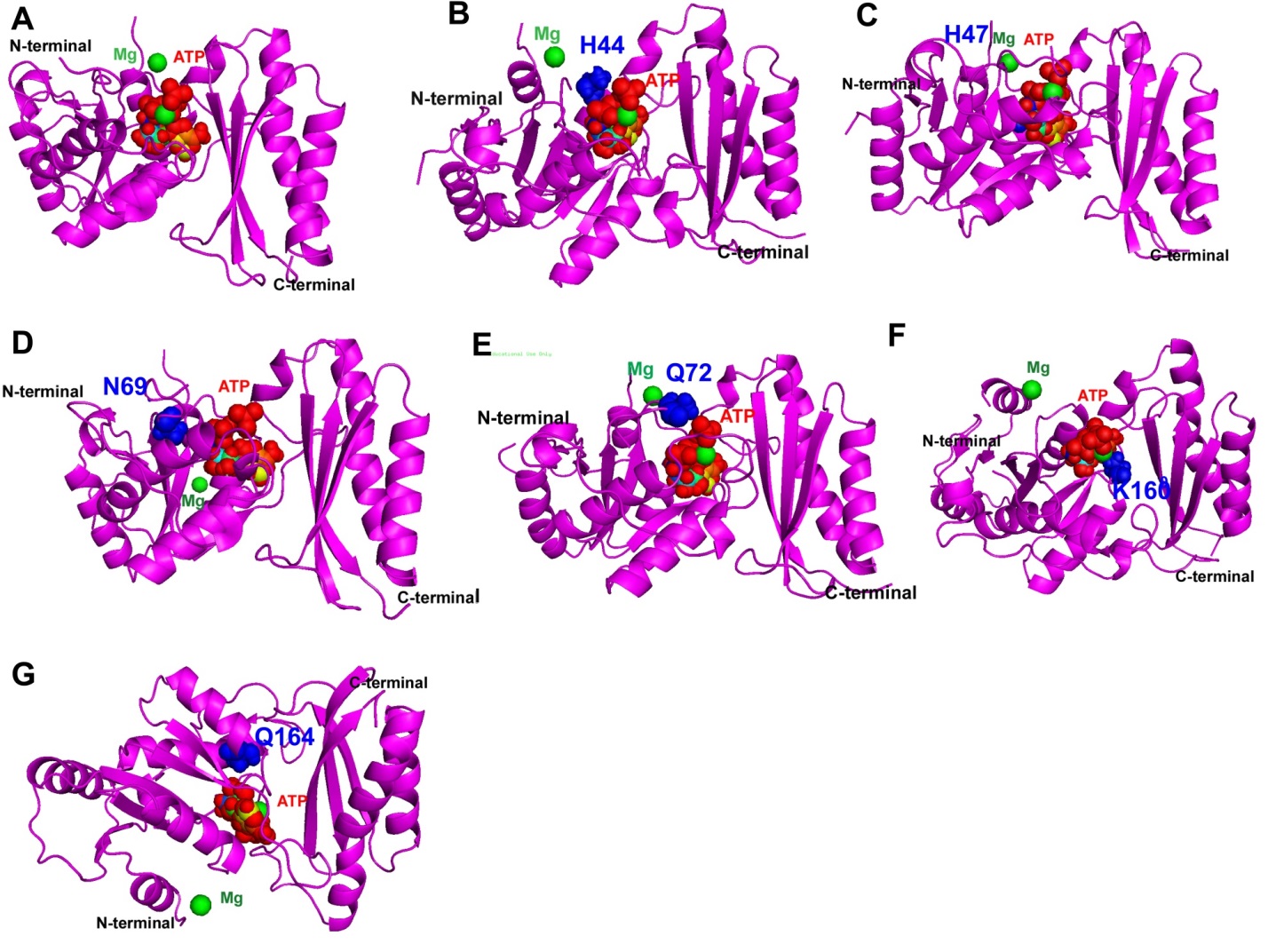


**Figure S2:** Conformational changes in the ATP binding in MD simulated (A) wild-type, (B) H44A, (C) H47A, (D) N69A, (E) Q72A, (F) K160A, (G) Q164A alanine mutated complexes.

**
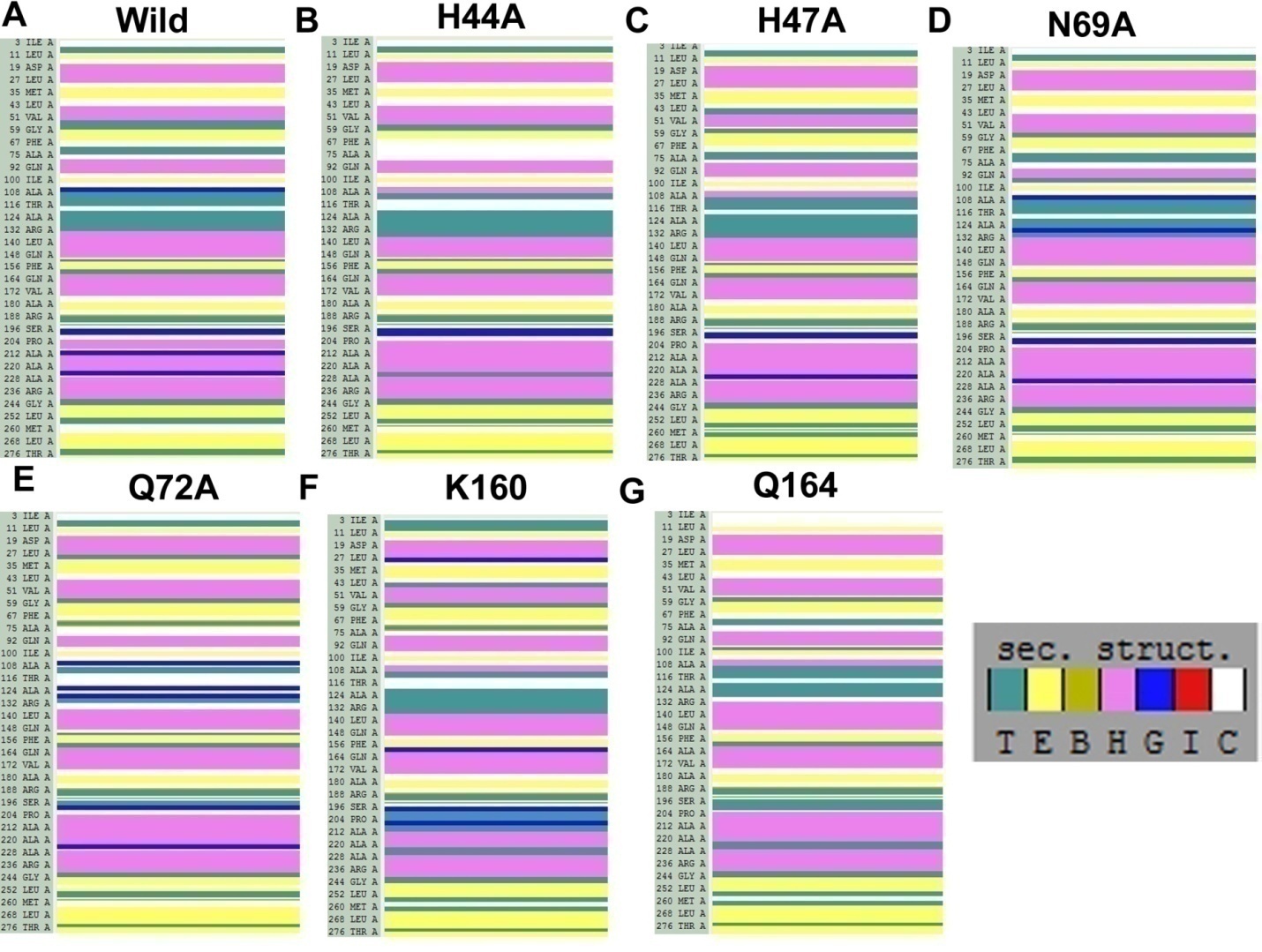
**

**Figure S3:** Secondary structure was computed for wild and alanine mutants. Where T, E, B, H, G, I, C represents turn, extended conformation (β-sheets), Isolated bridge, α-helix, 3-10 helix, Pi helix, and Coil respectively.

**
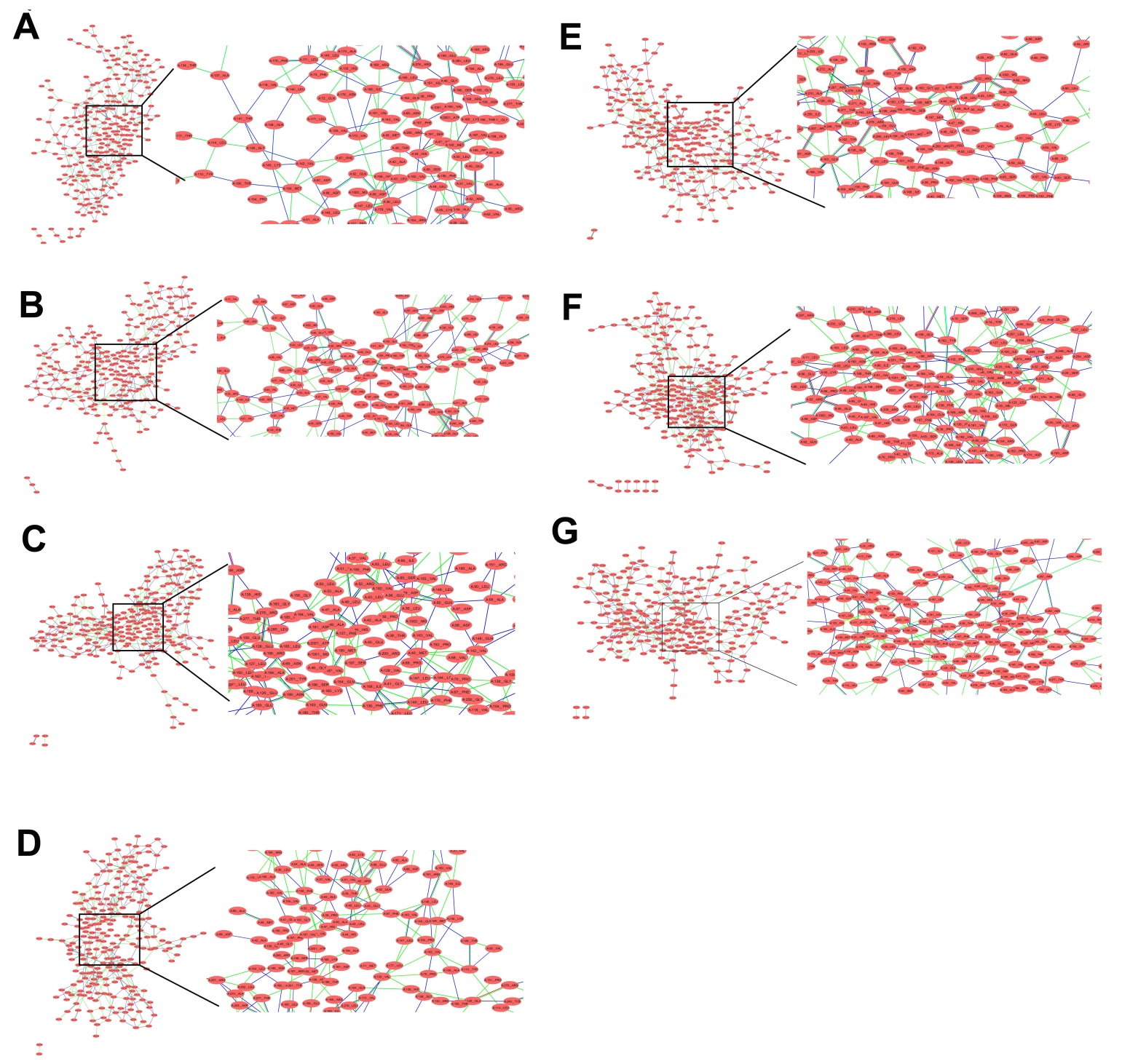
**

**Figure S4:** Comparative residue interaction diagram for (A) wild-type, (B) H44A, (C) H47A, (D) N69A, (E) Q72A, (F) K160A, (G) Q164A mutants for the conserved active site residues. Blue, red, yellow, violet and green color represents hydrogen bonds, ionic, cation-pi, pi-pi and vander waal force of interaction respectively.

**
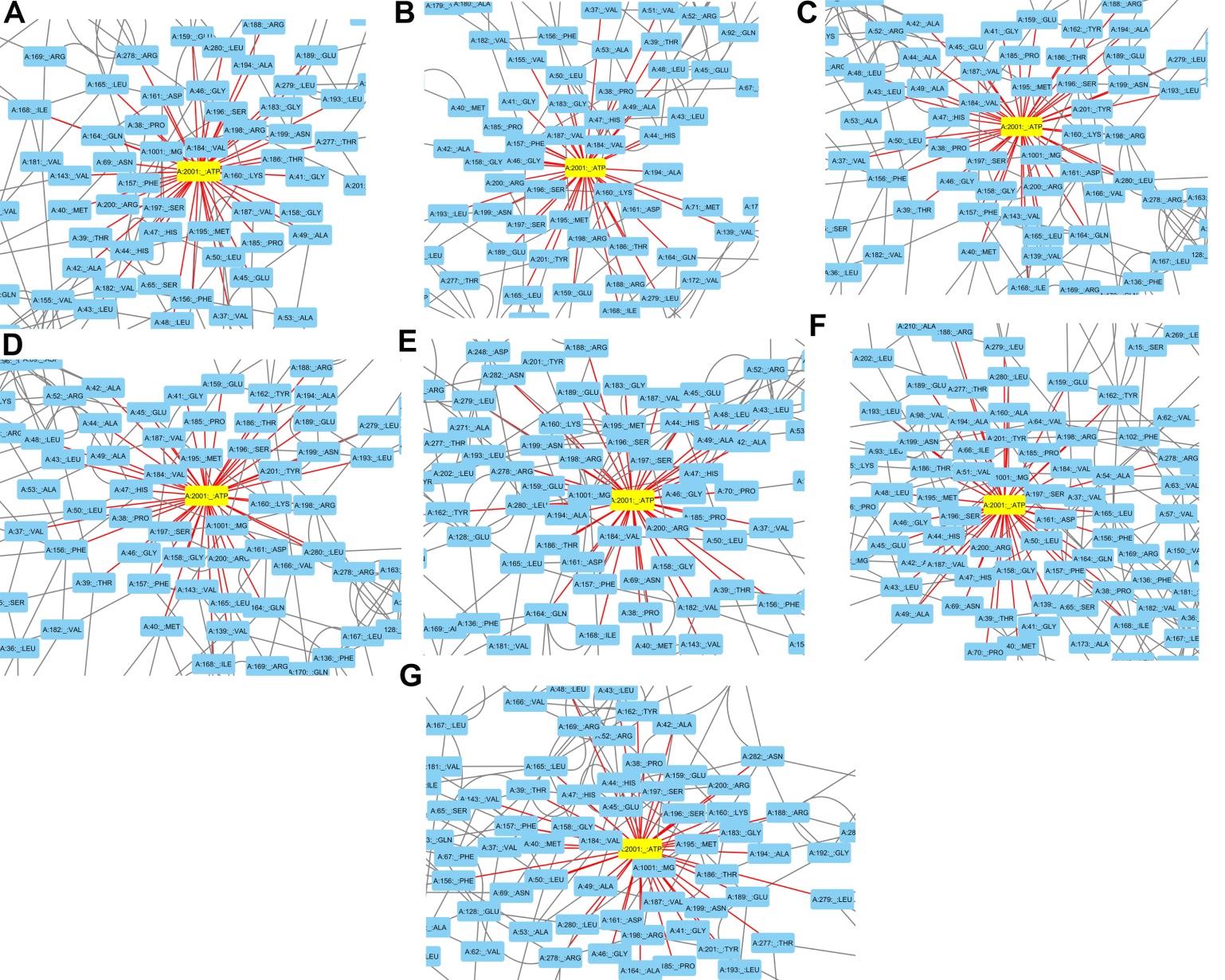
**

**Figure S5:** Network showing interaction between PS residues and ATP (A) wild-type, (B) H44A, (C) H47A, (D) N69A, (E) Q72A, (F) K160A, (G) Q164A mutants. Yellow color represents ATP and red lines shows the interaction between ATP and amino acid residues of PS.

**Table S1:** Interaction profile between PS and ATP in wild and alanine mutants before MD simulations.

| **Systems** | **No. of hydrogen bond** | **Participating residues with their bond length (Å)** | **Participating residues in hydrophobic bonding** | **Salt bridge interaction** |
| --- | --- | --- | --- | --- |
| **Wild-type** | 17 | Met40(2.8Å), Arg44(2.8Å), Arg47(2.9Å), Gly158(2.9Å, 3.2Å), Lys160(2.8Å, 2.9Å), Asp161(2.6Å), Val187(2.9Å, 2.9Å), Met195(2.8Å), Ser196(2.6Å, 2.6Å ), Ser197(2.8Å), Arg198(2.9Å, 2.9Å, 2.9Å) | Pro38, Thr39, Leu50, Gly146, Phe157, Thr186, Pro185 | Lys160, Arg198 |
| **H44A** | 15 | Met40(2.8Å), Arg47(2.9Å),Gly158(2.9Å, 3.2Å), Lys160(2.9Å, 2.8Å), Val187(2.9Å, 2.9Å), Met195(2.8Å,3.3Å), Ser196(2.6Å), Ser197(2.8Å), Arg198(2.9Å, 2.9Å, 2.9Å) | Pro38, Thr39, Gly46, Leu50, Phe157, Asp161, Thr186, Pro185 | Lys160, Arg198 |
| **H47A** | 15 | Met40(2.8Å), Arg44(2.8Å), Gly158(2.9Å, 3.2Å), Lys160(2.9Å, 2.8Å), Val187(2.9Å, 2.9Å), Met195(2.8Å,3.3Å), Ser196(2.6Å), Ser197(2.8Å), Arg198(2.9Å, 2.9Å, 2.9Å) | Pro38, Thr39, Leu50, Gly146, Phe157, Asp161, Thr186, Pro185 | Lys160, Arg198 |
| **N69A** | 13 | Met40(2.8Å), Arg44(2.8Å), Arg47(2.9Å), Gly158(2.9Å, 3.2Å), Lys160(2.8Å, 2.9Å), Val187(2.9Å, 2.9Å), Met195(2.8Å, 3.3), Ser196(2.6Å), Ser197(2.8Å) | Pro38, Thr39, Leu50, Gly146, Phe157, Asp161, Thr186, Pro185 | Lys160, Arg198 |
| **Q72A** | 16 | Met40(2.8Å), Arg44(2.8Å), Arg47(2.9Å), Gly158(2.9Å, 3.2Å), Lys160(2.9Å, 2.8Å), Val187(2.9Å, 2.9Å), Met195(2.8Å,3.3Å), Ser196(2.6Å), Ser197(2.8Å), Arg198(2.9Å, 2.9Å, 2.9Å) | Pro38, Thr39, Leu50, Gly146, Phe157, Asp161, Thr186, Pro185 | Lys160, Arg198 |
| **K160A** | 14 | Met40(2.8Å), Arg44(2.8Å), Arg47(2.9Å), Gly158(2.9Å, 3.2Å), Val187(2.9Å, 2.9Å), Met195(2.8Å,3.3Å), Ser196(2.6Å), Ser197(2.8Å), Arg198(2.9Å, 2.9Å, 2.9Å) | Pro38, Thr39, Leu50, Gly146, Phe157, Asp161, Thr186, Pro185 | Lys160, Arg198 |
| **Q164A** | 15 | Met40(2.8Å), Arg44(2.8Å), Arg47(2.9Å), Gly158(2.9Å, 3.2Å), Lys160(2.8Å, 2.9Å),, Val187(2.9Å, 2.9Å), Met195(2.8Å), Ser196(2.6Å, 2.6Å ), Ser197(2.8Å), Arg198(2.9Å, 2.9Å, 2.9Å) | Pro38, Thr39, Leu50, Gly146, Phe157, Asp161, Thr186, Pro185 | Lys160, Arg198 |

**Table S2:** Comparative secondary structure analysis of the wild and alanine mutants.

| **Systems** | **Coil**  **(C )**% | **Turn (T)**% | **α- Helix (H)**% | **β- Sheet**  **(E)**% | **3_10_-Helix**  **(G)**% | **Isolated βbridge**  **(B)**% |
| --- | --- | --- | --- | --- | --- | --- |
| **Wild-type** | 19.71 | 21.50 | 32.61 | 20.76 | 4.71 | 0.71 |
| **H44A** | 23.38 | 17.99 | 35.25 | 20.50 | 1.80 | 1.08 |
| **H47A** | 17.20 | 21.50 | 35.12 | 22.68 | 2.5 | 1.0 |
| **N69A** | 19.71 | 18.99 | 34.78 | 20.80 | 4.65 | 1.07 |
| **Q72A** | 24.01 | 14.33 | 32.61 | 21.89 | 5.37 | 1.79 |
| **K160A** | 18.99 | 25.44 | 30.10 | 22.25 | 2.15 | 1.07 |
| **Q164A** | 19.71 | 21.14 | 36.64 | 21.80 | - | 0.71 |

**Table S3:** List of residue interacting with ATP in residue interaction network.

| **Systems** | **Participating residues with their bond length <6Å** |
| --- | --- |
| **Wild-type** | Val37 (4.7), Pro38(2.4), Met40 (3.2), Gly41(3.6), Ala42(5.9), Leu43(4.9), His44(1.7), Glu45(6.0), Gly46(3.7), His47(3.2), Leu48(6.0), Ala49(4.3), Leu50(4.3), Asn69(5.5), Phe156(4.3), Phe157(2.3), Gly158(1.8), Glu159(3.5), Lys160(3.2), Asp161(3.5), Gln164(5.4), Val184(2.6), Pro185(2.7), Thr186(2.5), Val187(1.7), Arg188(4.1), Ala194(4.3), Mat195(2.0), Ser196(1.8), Ser197(1.7), Arg198(1.5), Asn199(4.5), Leu280(5.7) |
| **H44A** | Val37 (5.4), Pro38(2.1), Thr39(2.7), Met40 (4.1), Gly41(4.8), Ala42(5.6), Leu43(5.5), Ala44(5.0), Gly46(3.3), His47(3.5), Ala49(4.4), Leu50(2.3), Val139(5.3), Phe156(5.7), Phe157(2.7), Gly158(2.5), Glu159(4.2), Lys160(3.5), Asp161(1.6), Tyr162(5.8), Gln164(5.8), Ile168(5.8), Val184(3.3), Pro185(2.5), Thr186(2.5), Val187(1.7), Arg188(4.0), Leu193(6.0), Ala194(3.7), Mat195(1.9), Ser196(1.8), Ser197(2.0), Arg198(1.5), Asn199(4.8), Arg200(5.7), Tyr201(5.5), Leu280(5.3) |
| **H47A** | Val37 (5.6), Pro38(2.1), Thr39(3.9), Met40 (2.1), Gly41(5.4), His44(4.0), Glu45(4.0), Gly46(3.7), Ala47(2.6), Leu48(5.8), Ala49(5.0), Leu50(2.4), Asn69(6.0), Phe157(2.9), Gly158(2.0), Glu159(4.1), Lys160(3.6), Asp161(2.5), Gln164(4.4), Leu165(5.9),Val184(3.4), Pro185(2.5), Thr186(2.5), Val187(1.9), Arg188(4.2), Ala194(3.9), Met195(1.9), Ser196(1.8), Ser197(2.7), Arg198(15), Arg280(5.9) |
| **N69A** | Val37 (4.4), Pro38(2.8), Thr39(3.4), Met40 (3.0), Gly41(4.1), Ala42(5.8), Leu43(5.2), His44(1.6), Gly46(3.6), His47(2.4), Ala49(5.6), Leu50(3.8), Mat71(5.2), Phe156(4.8), Phe157(3.0), Gly158(2.3), Glu159(3.1), Lys160(3.7), Asp161(3.7), Val184(3.2), Pro185(3.8), Thr186(2.3), Val187(2.0), Arg188(4.2), Leu193(5.9), Ala194(2.9), Mat195(2.0), Ser196(1.6), Ser197(1.7), Arg198(1.5), Asn199(4.1) |
| **Q72A** | Val37 (5.0), Pro38(4.3), Thr39(3.3), Met40 (4.1), His44(1.7), Glu45(5.6), Gly46(3.7), His47(3.3), Ala49(4.5), Leu50(2.4), Asn69(5.6), Phe156(5.3), Phe157(2.1), Gly158(2.0), Glu159(3.8), Lys160(3.6), Asp161(2.0), Tyr162(5.0), Gln164(4.9), Leu165(4.7), Ile168(5.1), Val184(2.9), Pro185(2.8), Thr186(2.9), Val187(1.8), Arg188(3.9), Glu189(5.9), Ala194(4.1), Mat195(1.8), Ser196(1.7), Ser197(1.9), Arg198(1.7), Asn199(4.4) |
| **K160A** | Pro38(4.9), Thr39(4.5), Met40 (4.0), Gly41(5.5), Ala42(5.8), Leu43(5.6), His44(1.6), Gly46(3.9), His47(2.7), Leu48(5.8), Ala49(4.8), Leu50(2.4), Asn69(3.5), Phe157(3.2), Gly158(2.4), Glu159(3.6), Lys160(3.8), Asp161(1.8), Gln164(5.1), Val184(4.0), Pro185(2.8), Thr186(2.5), Val187(4.0), Arg188(4.2), Ala194(3.3), Mat195(1.7), Ser196(2.3), Ser197(1.5), Arg198(1.7), Asn199(5.0), Tyr201(4.0), Thr277(5.7), Leu280(4.9) |
| **Q164A** | Val37 (5.1),Pro38(2.4), Thr39(4.0), Met40 (2.5), Gly41(3.5), Ala42(4.8), His44(3.9), Gly46(3.8), His47(3.0), Ala49(4.9), Leu50(2.4), Asn69(3.5), Phe156(5.5), Phe157(2.1), Gly158(1.9), Glu159(3.5), Ala160(3.5), Asp161(2.3), Tyr162(4.8), Ala164(4.5), Leu165(4.4), Val184(3.0), Pro185(2.8), Thr186(2.5), Val187(1.7), Arg188(3.9), Glu189(5.7), Leu193(5.6), Ala194(3.5), Mat195(2.0), Ser196(1.6), Ser197(1.8), Arg198(1.5), Asn199(4.3), Arg200(6.0), Tyr201(5.5), Leu280(5.7) |
